# Prognostic Factors for the Therapeutic Performance of Cisplatin in Head and Neck Malignancies

**DOI:** 10.3389/fonc.2022.778380

**Published:** 2022-04-28

**Authors:** Frederic Jungbauer, Lena Huber, Sonja Ludwig, Nicole Rotter, Beatrice Walter, Lena Zaubitzer, Anne Lammert

**Affiliations:** Department for Otorhinolaryngology, Head- and Neck-Surgery, University Medical Centre Mannheim, Mannheim, Germany

**Keywords:** HNSCC, cisplatin, prognosis, laboratory tests, carcinoma, chemotherapy, predictive, blood values

## Abstract

**Introduction:**

For squamous cell carcinoma of the head and neck (HNSCC), cisplatin is used as primary or adjuvant (radio)chemotherapy. In terms of dosage, two main regimens are used, weekly 40mg/m^2^ or 3-weekly 100mg/m^2^. For an optimal outcome, the highest possible cumulative total dose of cisplatin is aimed for. The selection of the scheme is patient-specific, but the factors for the selection of the optimal scheme have not yet been conclusively researched. The aim of this study was to find correlations between initial laboratory values and the cumulative total dose of cisplatin, as well as any correlations between early laboratory values or their dynamics and later laboratory values or their dynamics to provide support in the selection of the chemo regimen.

**Material and Methods:**

In this retrospective study, the clinical data and laboratory values, namely glomerular filtration rate (GFR), hemoglobin, albumin, leucocyte, erythrocyte and platelet count, over the course of time of 79 patients with HNSCC who had received chemotherapy with cisplatin in our clinic between 2018 and 2021 were evaluated.

**Results:**

Patients on 3-weekly regimens achieved a higher mean cumulative total dose of cisplatin than patients on weekly regimens (214.18 ± 65.95 vs 183.33 ± 65.2 mg/m^2^). Significant positive correlations were seen for total cumulative dose of cisplatin with initial GFR (p=0.001, Pearson’s r=0.364), initial hemoglobin (p=0.035, r=0.237), initial erythrocyte (p=0.002, r=0.337), and initial albumin (p=0.002, r=0.337). There were no significant correlations for initial leucocyte or platelets. Regarding the dynamics of the laboratory values under the first chemo administration, no correlation was found with later laboratory values or dynamics.

**Discussion and Conclusion:**

As in other prospective studies, our retrospective analysis found a higher cumulative total dose in the 3-weekly regimen. As this seems to correlate positively with patient outcome, superiority of the 3-weekly regimen over the weekly regimen can be assumed. Functioning organ systems, especially of the bone marrow and kidneys, are associated with an increased cumulative total dose and can therefore be regarded as predictive factors. Regular monitoring of laboratory values is nevertheless essential throughout the entire course of chemotherapy.

## Introduction

Cisplatin is an inorganic heavy metal complex with the molecular formula Cl2H6N2Pt. It was the first platinum-containing agent approved by the United States Food and Drug Administration for the treatment of cancer in 1978 (1). Intracellularly, cisplatin loses its chloride ions, creating a reactive species that generates linkage with the purine bases of DNA (2). Cross-linking sets DNA damage, which subsequently leads to apoptosis of the affected cell through various signal transduction pathways (3). Due to increasing resistance mechanisms to cisplatin, the underlying molecular mechanisms continue to be the focus of oncology research (4).

In the treatment of squamous cell carcinoma of the head and neck region (HNSCC), cisplatin is used both as part of primary (without previous surgery) and adjuvant (after previous surgery) radiochemotherapy (pRCT/aRCT). Possible applications have also been established in combination with other antineoplastic agents such as cetuximab or 5-fluorouracil (5-FU), as in the EXTREME regimen (5). Other platinum derivatives such as carboplatin can also be used, but because of the superiority of cisplatin, they are usually chosen only when, because of individual risk factors, therapy with cisplatin appears too risky. This may be the case, for example, if kidney function is too weak at the beginning or during the course of chemotherapy (6, 7).

The main known side effects of cisplatin are a high emetogenic potential and various organotoxic effects. Cisplatin can be nephrotoxic (8), ototoxic (9) and neurotoxic (especially with peripheral neuropathies) (10) as well as myelosuppressive (11). Appropriate premedication with e.g. cortisone, histamine antagonists and 5-hydroxytryptamine antagonists should keep nausea within tolerable limits. Because of the possible organ damage mentioned, laboratory-chemical blood checks must be carried out before and after each administration in order to be able to recognize and treat possible complications. If there are signs of incipient organ damage, an individual decision must be made as to whether chemotherapy can be continued with supportive measures, whether it must be paused, whether a switch to another agent must be made, or whether chemotherapy must be discontinued completely. Most common reasons for discontinuation or switching from cisplatin to another agent appear to be nephrotoxic and myelosuppressive effects (12). However, other factors can also result in the discontinuation of therapy, such as serious infections or even the patient’s refusal to continue therapy.

Studies have shown that the outcome of patients after pRCT and aRCT depends mainly on the cumulative total dose, i.e. the added dose of all administrations of cisplatin in the course of therapy. The higher the total cumulative dose achieved, the better the outcome of patients (13). Thus, the goal of RCT is to administer as high a dose of cisplatin as possible during ongoing radiotherapy, while preserving organ function and reducing toxicity and side effects as much as possible. Various regimens are used for this purpose. The two most common are weekly administration à 40mg cisplatin/m^2^ body surface area and 3-weekly administration à 100mg cisplatin/m^2^ body surface area intravenously during radiotherapy (14). Currently, there are no uniform recommendations as to which therapy regimen should be selected. On the one hand, patients seem to achieve better locoregional control with the 3-weekly regimen, but on the other hand, the higher tumor toxicity also leads to more pronounced side effects than with the weekly regimen (15, 16).

The aim of this study was to demonstrate possible correlations of early laboratory chemical changes with the cumulative total dose achieved later and to be able to provide support for the selection of the cisplatin regimen based on subgroup analyses.

## Material and Methods

All patients who had a presentation to the Interdisciplinary Head and Neck Tumor Board of the University Medical Centre Mannheim (Mannheim, Germany) from 01/2018 to 05/2021 were screened. Screening was performed only through 2018 because prior to that, cisplatin was administered mostly in combination with 5-FU, not as monotherapy. Radiation fractionation of 2Gy per day was performed in both adjuvant and primary RCT. Thus, a total cumulative dose of 60Gy was achieved at the completion of therapy. In the primary RCT, if therapy could be completed, a subsequent boost of 10Gy was applied to the tumor region.

Patients who received curative therapy and started either primary radiochemotherapy with cisplatin without another chemotherapy agent or adjuvant radiochemotherapy with cisplatin without another chemotherapy agent were included. Patients on other platinum-based chemotherapies (e.g., carboplatin), combined chemotherapeutics (e.g., cisplatin/5-FU), or palliative regimens (e.g., the EXTREME regimen with platinum/cetuximab) were not included. Clinical data such as patient age, cisplatin regimen used, and any port or percutaneous endoscopic gastrostomy (PEG) implantation were extracted from medical records. Blood sampling was done on the day of chemo administration or the day before. The control of blood values after chemotherapy administration was performed 3-7 days after chemotherapy administration. Blood values were extracted from the in-house laboratory system before and after the respective chemo administrations, namely leukocyte count, glomerular filtration rate (GFR, calculated from serum creatinine), platelet count, hemoglobin, erythrocyte count, and serum albumin. In addition to the enumerated values, their differences between before and after chemo administration, both in absolute and relative values, were also calculated.

Statistical analysis was then performed using the statistical software SPSS Statistics for Windows, version 27.0 (SPSS Inc., Chicago, Ill., USA). Descriptive analyses, t-tests, and bivariate correlations were performed. A p-value <0.05 was considered statistically significant. The results are given in absolute numbers ± standard deviation. A professional consultation took place by the local ethics committee of the University of Heidelberg and did not result in any concerns (approval number 2021-865).

## Results

Of the patients screened, 79 patients (n=79) were included. All patients had squamous cell carcinoma on histopathology, and the location, tumor extent, lymph node status, and distant metastasis status (according to the respective Union for International Cancer Control TNM classification) are listed in **Table 1**. The mean age was 63 years (± 9 years, range 33-80 years). 64 patients (81%) were male, 15 (19%) were female. Radiochemotherapy was started adjuvantly (after a previous resectioning surgery) in 31 patients (39.2%) and as primary/definitive therapy (without previous surgery) in 48 patients (60.8%). Chemotherapy with weekly regimen was started in 30 patients, 3-weekly regimen in 49 patients (38%/62%). A PEG was placed in 46 patients (58.2%). A port was implanted in 28 patients (35.4%). The first dose of cisplatin 3-weekly was given in 49 patients (62%), a second dose of cisplatin 3-weekly in 37 patients (47%), and a third dose of cisplatin 3-weekly in 12 patients (15%). Due to adverse events, the regimen was changed from 3-weekly to weekly in 4 patients (5%) after the first administration. The first dose of cisplatin weekly was given in 30 patients (38%), a second dose of cisplatin weekly in 32 patients (41%), a third dose of cisplatin weekly in 29 patients (37%), a fourth dose of cisplatin weekly in 25 patients (32%), a fifth dose of cisplatin weekly in 19 patients (24%), a sixth dose of cisplatin weekly in 12 patients (15%), and seven doses of cisplatin weekly in 2 patients (2.5%).

**Table 1:** Characteristics and initial laboratory values of the examined patients (T, tumor extent; N, lymph node status; M, distant metastasis status).

ID	Sex [male; female]	Age at start of chemotherapy [years]	T	N	M	Localization	Adj./prim. RCT	Initial regimen cisplatin [mg/m^2]	Accumulated dose cisplatin [mg/m^2]	Death during the study period	Initial leucocyte count [10E9/L]	Initial GFR [ml/min/1,73m^2]	Initial platelet count [10E9/L]	Initial hemoglobin [g/dL]	Initial erythrocyte count [10E12/L]	Initial albumin [g/L]
1	m	64	2	1	0	oropharynx	adj.	100	300	no	5.98	94	216	13.7	4.73	38
2	m	59	4	1	0	oral cavity / oropharynx	prim.	100	200	no	7.58	101	347	15.7	5.15	44
3	m	61	4	0	0	oral cavity	prim.	40	40	no	4.04	76	138	9.2	2.79	30.8
4	m	49	2	1	0	oropharynx	adj.	100	200	no	7.24	99	436	11.5	4	34
5	m	79	2	2	0	larynx	prim.	40	40	no	8.21	52	195	15	4.75	36.5
6	m	73	3	0	0	hypopharynx	prim.	40	160	no	6.41	73	191	14	4.25	34.8
7	m	60	3	0	0	oropharynx / hypopharynx	adj.	100	200	no	5.91	104	463	9.5	3.25	38
8	f	74	4	2	0	oropharynx / hypopharynx / larynx	prim.	40	200	no	5.5	92	240	12.9	3.9	37
9	m	50	4	3	1	larynx	adj.	100	260	no	8.35	106	631	9.8	3.4	36
10	m	53	3	2	0	oropharynx	adj.	100	300	no	8.58	111	407	12.5	4.31	37
11	m	64	3	1	0	hypopharynx / larynx	prim.	100	100	no	4.97	73	293	12.6	4.29	39
12	m	51	1	2	0	oropharynx	adj.	100	180	no	10.24	115	461	11.7	3.67	32
13	m	63	3	2	0	hypopharynx	prim.	100	300	no	8.22	85	169	14.1	5.24	35.7
14	f	59	4	1	0	nasopharynx	prim.	100	200	?	16.95	92	474	11.5	4.03	32.3
15	m	74	2	1	0	oropharynx / hypopharynx	adj.	100	200	no	6.38	82	506	11.9	3.83	28.8
16	m	58	3	3	0	hypopharynx	adj.	100	220	no	9.57	98	427	10.9	3.91	34
17	m	39	4	0	0	oropharynx	prim.	100	250	no	9.77	118	189	14.2	4.68	37
18	f	53	4	0	0	nasopharynx / oropharynx	prim.	100	200	no	7.1	109	323	13.3	4.05	31
19	f	64	4	2	0	nasopharynx / oropharynx / hypopharynx	prim.	40	160	yes	14.46	95	599	7.3	3.17	23
20	m	55	1	1	0	nasopharynx	prim.	100	300	no	5.43	94	268	14.5	4.85	36.8
21	f	59	1	1	0	oropharynx	adj.	100	100	no	9.92	96	254	12.8	4.01	35
22	f	74	3	2	0	oropharynx / larynx	prim.	40	120	yes	9.66	68	276	13.2	4.38	34
23	m	71	3	2	0	oral cavity	adj.	40	200	no	4.84	90	416	12.6	3.88	29.3
24	m	46	4	2	0	oral cavity	prim.	40	160	no	7.97	100	367	12.3	4.55	35.7
25	m	54	4	2	0	oropharynx	adj.	100	260	yes	8.89	84	389	13.7	4.79	36.3
26	m	69	3	2	0	oropharynx	adj.	40	160	no	8.79	85	286	12.5	4.07	29.8
27	m	71	4	1	0	oropharynx	prim.	40	80	yes	9.78	62	252	8.1	2.54	25.6
28	m	71	4	0	0	hypopharynx	prim.	40	240	no	9.11	70	391	13.6	3.85	33.1
29	m	65	1	0	0	larynx	prim.	40	240	no	6.07	90	213	16.4	5.28	36
30	m	72	3	0	0	larynx	prim.	40	240	no	6.54	67	278	14.6	4.66	37.2
31	m	64	2	3	0	hypopharynx	adj.	100	250	no	8.84	79	169	13.1	4.45	38.3
32	f	78	1	2	0	oropharynx	prim.	40	240	no	6.97	65	359	13.5	4.52	36
33	m	54	3	0	0	oropharynx	prim.	100	300	yes	8.06	102	309	12.7	3.94	33.8
34	m	33	2	1	0	oral cavity	adj.	100	200	no	7.91	125	364	11.4	3.69	32.3
35	f	77	3	2	0	oropharynx	prim.	40	240	no	5.98	75	284	14.2	4.66	28.4
36	m	57	4	0	0	nasopharynx	prim.	100	300	no	8.5	65	522	8.7	4.36	28.1
37	m	59	1	2	0	oropharynx	prim.	100	200	no	6.11	87	150	11.6	4	38
38	f	53	1	1	0	oropharynx	adj.	100	200	no	7.09	77	426	14	4.95	38.2
39	m	66	3	2	0	oropharynx	prim.	100	100	no	9.87	73	299	15.9	5.33	36.6
40	m	72	3	0	0	hypopharynx	prim.	100	100	no	8.28	61	236	14.3	4.27	37.5
41	m	75	3	2	0	larynx	prim.	40	240	?	6.66	68	360	11.7	4.03	39.3
42	m	72	1	2	0	oral cavity	adj.	40	240	no	5.75	56	299	15.7	5.43	36.9
43	m	57	2	1	0	oropharynx	adj.	100	100	no	5.85	104	286	9.6	3.16	24.6
44	m	65	3	2	0	hypopharynx	prim.	40	200	no	7.97	74	236	13.9	4.58	37.2
45	m	61	4	2	0	nasopharynx / oropharynx	prim.	100	200	no	10.27	96	273	13.7	4.16	34.1
46	m	63	2	2	1	oral cavity	prim.	100	200	?	9	86	337	14	4.03	37.5
47	m	66	4	0	0	oropharynx	adj.	40	120	yes	7.15	86	679	11.6	4.07	28.7
48	f	68	1	1	0	nasopharynx	prim.	100	200	?	9.56	85	327	14.3	4.68	35.9
49	f	64	2	2	0	oral cavity	adj.	100	200	no	7.24	99	242	11.5	3.86	32.4
50	f	59	2	3	0	oropharynx	adj.	40	160	yes	9.26	95	455	11.1	3.36	25.8
51	m	64	4	2	0	oropharynx	prim.	40	200	no	9.59	73	349	12.6	4.17	35.1
52	m	55	2	1	0	oropharynx	adj.	100	200	no	6.12	96	273	12.1	7	37
53	f	74	4	0	0	larynx	prim.	40	260	yes	6.83	84	256	12.5	4.95	34.5
54	m	61	3	3	0	larynx	adj.	100	300	no	11.25	107	386	13.7	4.45	37.1
55	m	64	4	2	0	oropharynx	prim.	100	300	no	14.68	93	276	13.3	4.26	35.8
56	m	57	3	2	0	larynx	prim.	100	200	no	6.73	98	248	14.7	5.22	34.6
57	m	63	1	3	0	hypopharynx	adj.	100	200	no	6.02	101	427	10	3.46	28.2
58	m	72	3	0	0	larynx	prim.	40	240	?	11.6	91	360	12.8	4.12	35.5
59	m	64	2	1	0	oropharynx	adj.	100	100	?	7.54	96	417	10.8	3.46	30.1
60	m	57	3	1	0	hypopharynx	adj.	100	300	no	12.83	104	428	10.7	3.6	31.8
61	m	63	2	0	0	oral cavity	prim.	100	200	no	7.43	91	235	14.5	4.73	34.3
62	m	76	4	0	0	oropharynx	prim.	40	80	yes	9.22	78	539	9.5	3.07	18.4
63	m	57	3	2	0	larynx	prim.	100	200	?	10.11	110	575	12.4	3.97	28
64	m	59	4	2	0	oropharynx	prim.	40	240	?	7.65	117	420	12.2	3.83	32.5
65	m	70	1	2	0	oral cavity	adj.	100	100	no	5.72	68	378	11.8	3.82	31.4
66	m	71	4	3	0	oropharynx / hypopharynx	prim.	40	240	?	9.36	71	192	12.5	4.04	34.4
67	m	65	4	2	1	oropharynx / hypopharynx / larynx	prim.	100	200	yes	4.39	111	275	9.4	3.21	27.5
68	m	61	4	1	0	hypopharynx	prim.	100	300	?	8.93	95	375	15.4	5.03	37.6
69	m	50	4	0	0	oral cavity	prim.	100	300	?	9.25	121	344	11.7	4.41	33.5
70	m	70	2	2	0	oropharynx	prim.	40	160	no	5.45	70	166	11.5	3.63	34.7
71	m	47	3	0	0	larynx	adj.	100	200	?	5.06	102	116	13.2	4.65	35.2
72	f	72	3	1	0	oral cavity	adj.	100	200	yes	6.93	85	287	13.8	4.46	34.5
73	m	62	2	2	0	larynx	adj.	100	100	?	8.17	82	356	12.4	3.91	31.3
74	f	70	2	0	0	neopharynx	prim.	40	240	no	7.76	125	318	11	3.63	34.7
75	m	59	1	2	0	hypopharynx	adj.	100	300	no	7.37	99	442	13.3	4.5	33.4
76	m	80	2	1	0	oropharynx	prim.	40	120	no	5.05	53	200	12.3	3.97	31.8
77	m	61	2	0	0	oropharynx	prim.	100	275	no	7.23	90	250	15.3	5.26	37.8
78	m	68	3	2	0	oropharynx	prim.	40	240	yes	9.29	96	370	11.4	3.24	29.8
79	m	65	3	2	0	larynx	adj.	100	200	no	7.59	79	313	13.1	4.39	33.3

The total cumulative dose achieved was a mean of 202.47mg cisplatin/m^2^ body surface area (± 66.96; range 40-300). A dose of ≥200mg/m^2^ body surface area was achieved in 57 patients (72.51%). Here, a cumulative dose of >200mg/m^2^ body surface area was achieved within the weekly regimen group in 56.6% of patients, and within the 3-weekly regimen group in 81.6% of patients. The agent was switched from cisplatin to carboplatin in 8 patients (10.12%) during the course of therapy, and 2 patients (2.53%) were switched to primary radioimmunotherapy with cetuximab.

12 patients (15.2%) died during the observation period, 54 patients (68.3%) were alive at the end of the observation period. In 13 patients (16.5%), death within or survival of the observation period could not be traced on the basis of the available data. Of the 12 patients who died, 8 patients had started radiochemotherapy with the weekly regimen and 4 patients with the 3-weekly regimen. On average, the deceased patients initially had significantly higher platelet levels (p=0.039), significantly lower albumin levels (p<0.001), significantly lower erythrocyte levels (p=0.019), and significantly lower hemoglobin levels (p=0.008) than the survivors. There were no significant differences in leukocyte or GFR levels compared with patients who were alive at the end of the observation period.

**Figure 1:**
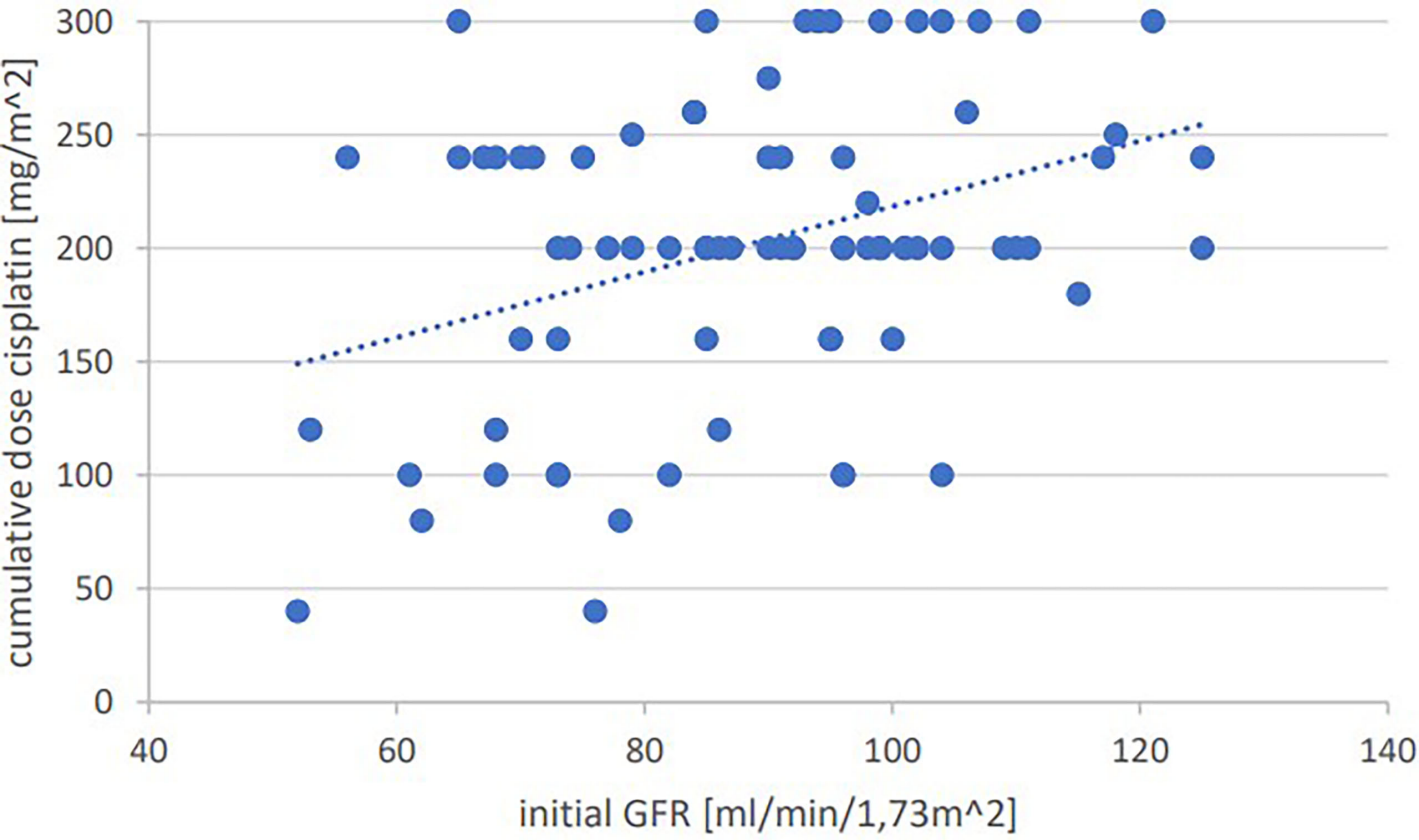
Bivariate correlation analysis of initial GFR values [ml/min/1,73m^2] with final cumulative total dose of cisplatin achieved [mg/m^2]: p=0.001, Pearson’s r=0.364; individual values as points, dashed linear trend line.

**Figure 2:**
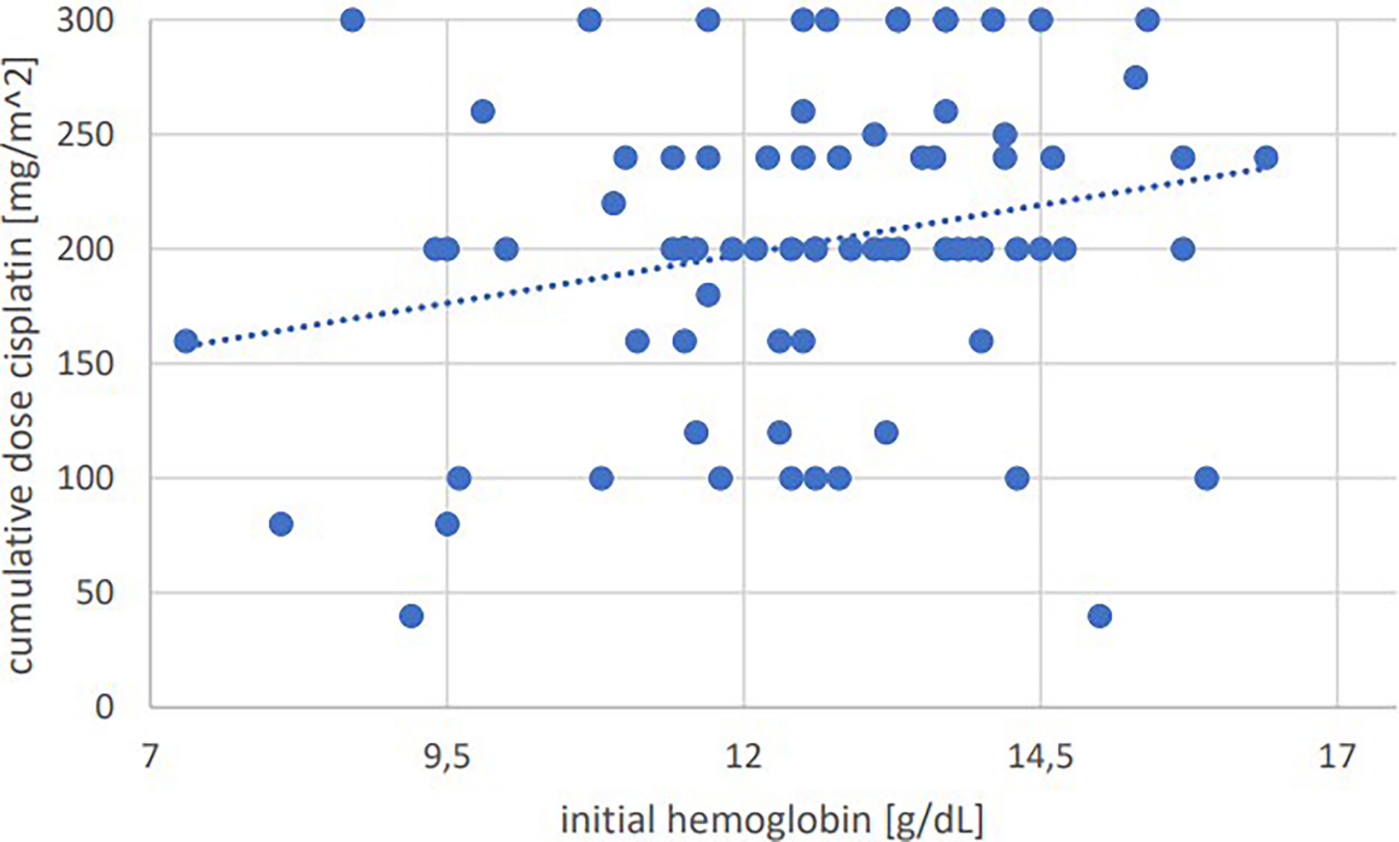
Bivariate correlation analysis of initial hemoglobin values [g/dL] with final cumulative total dose of cisplatin achieved [mg/m^2]: p=0.035, Pearson’s r=0.237; individual values as points, dashed linear trend line.

**Figure 3:**
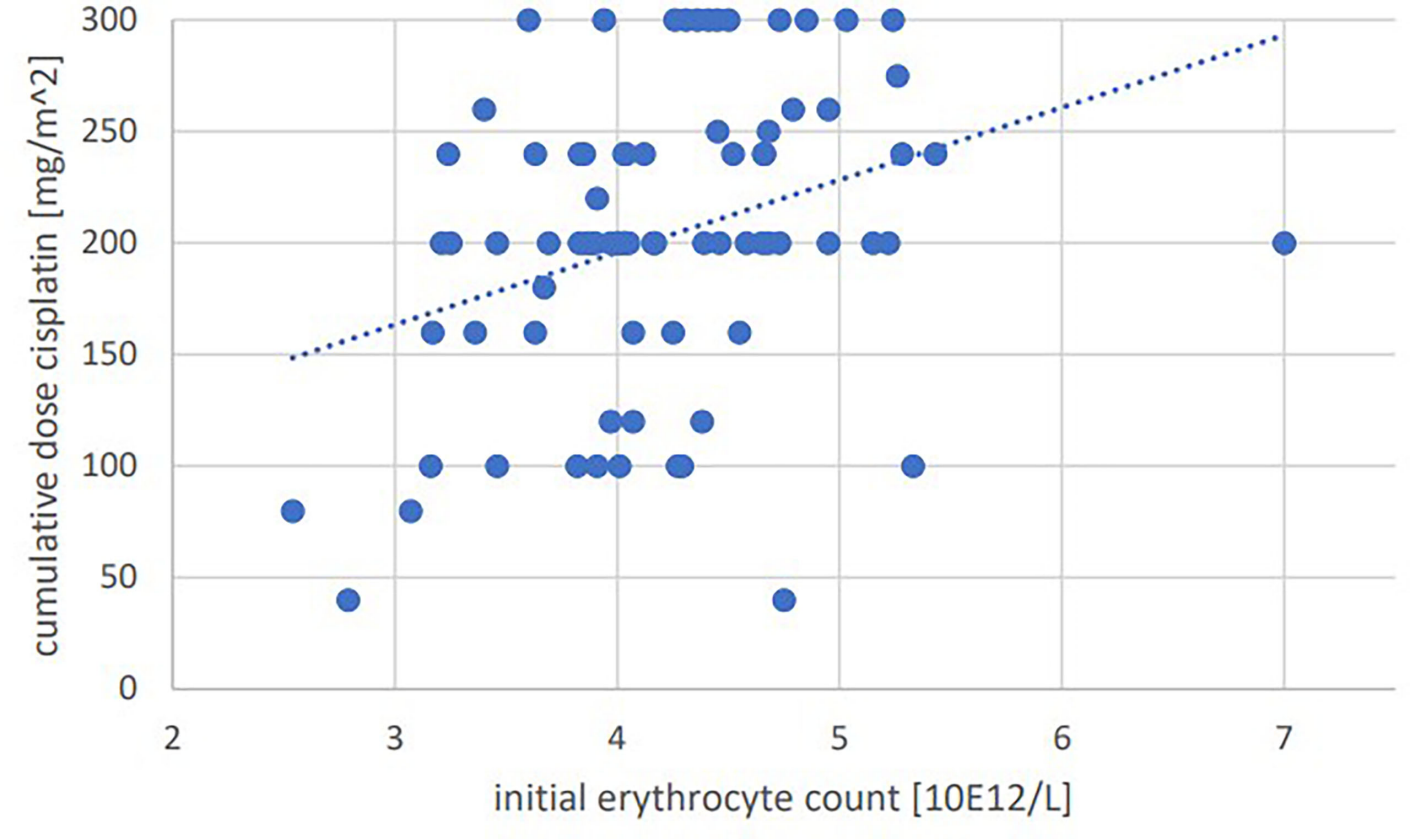
Bivariate correlation analysis of initial erythrocyte count [10E12/L] with final cumulative total dose of cisplatin achieved [mg/m^2]: p=0.002, Pearson’s r=0.337; individual values as points, dashed linear trend line.

**Figure 4:**
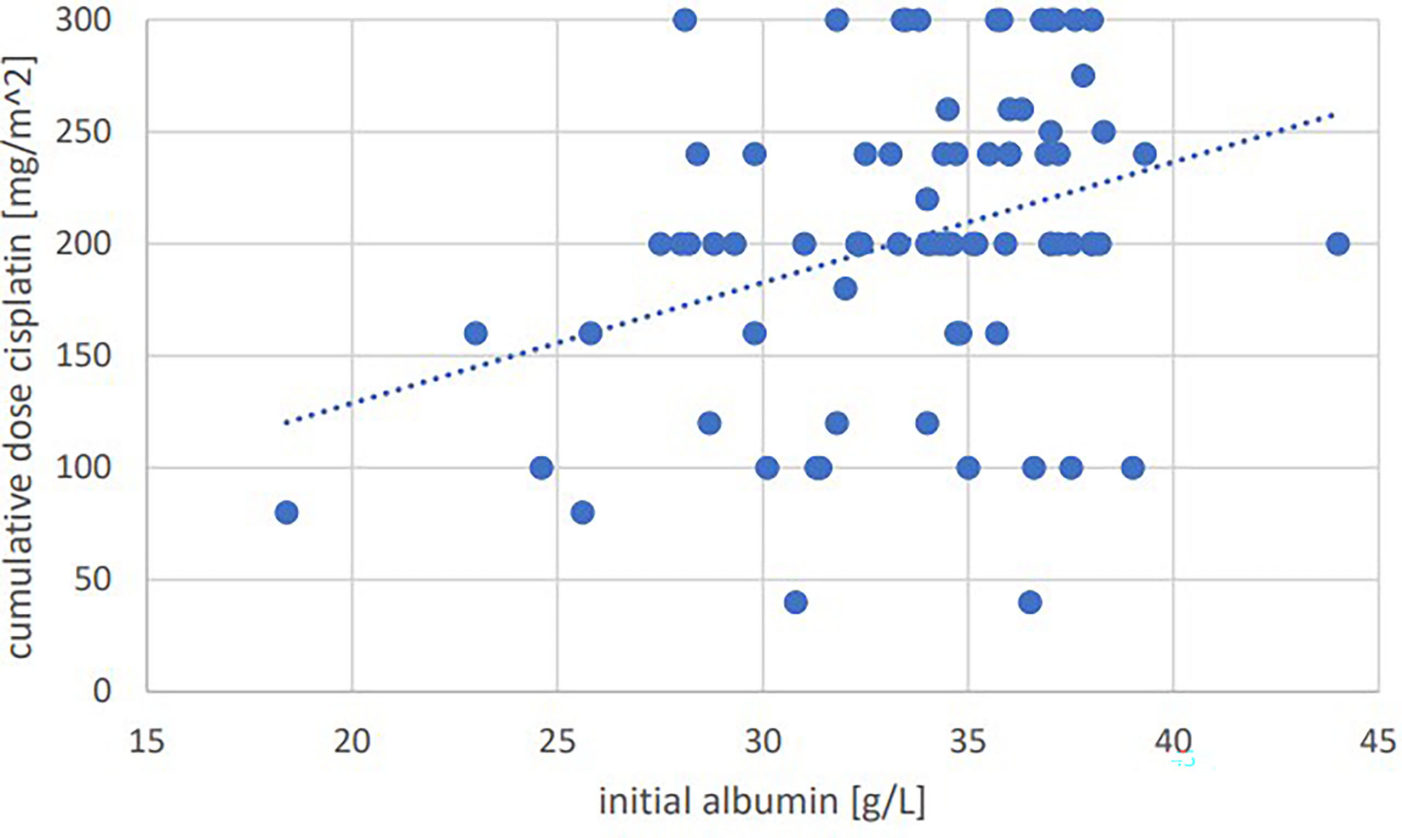
Bivariate correlation analysis of initial albumin value [g/L] with final cumulative total dose of cisplatin achieved [mg/m^2]: p=0.002, Pearson’s r=0.337; individual values as points, dashed linear trend line.

**Figure 5:**
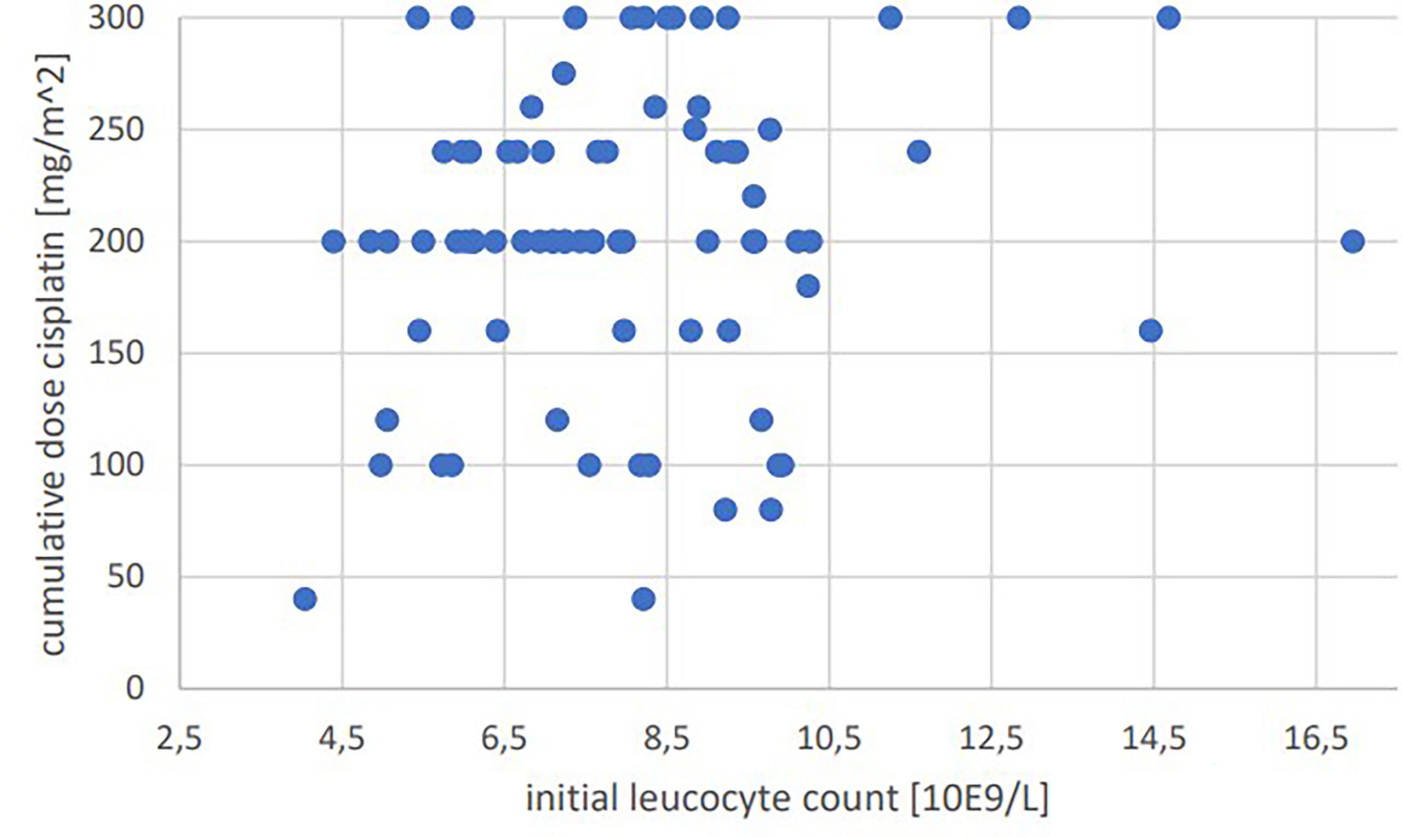
Bivariate correlation analysis of initial leucocyte count [10E9/L] with final cumulative total dose of cisplatin achieved [mg/m^2]: p=0.177, no significant correlation; individual values as points.

**Figure 6:**
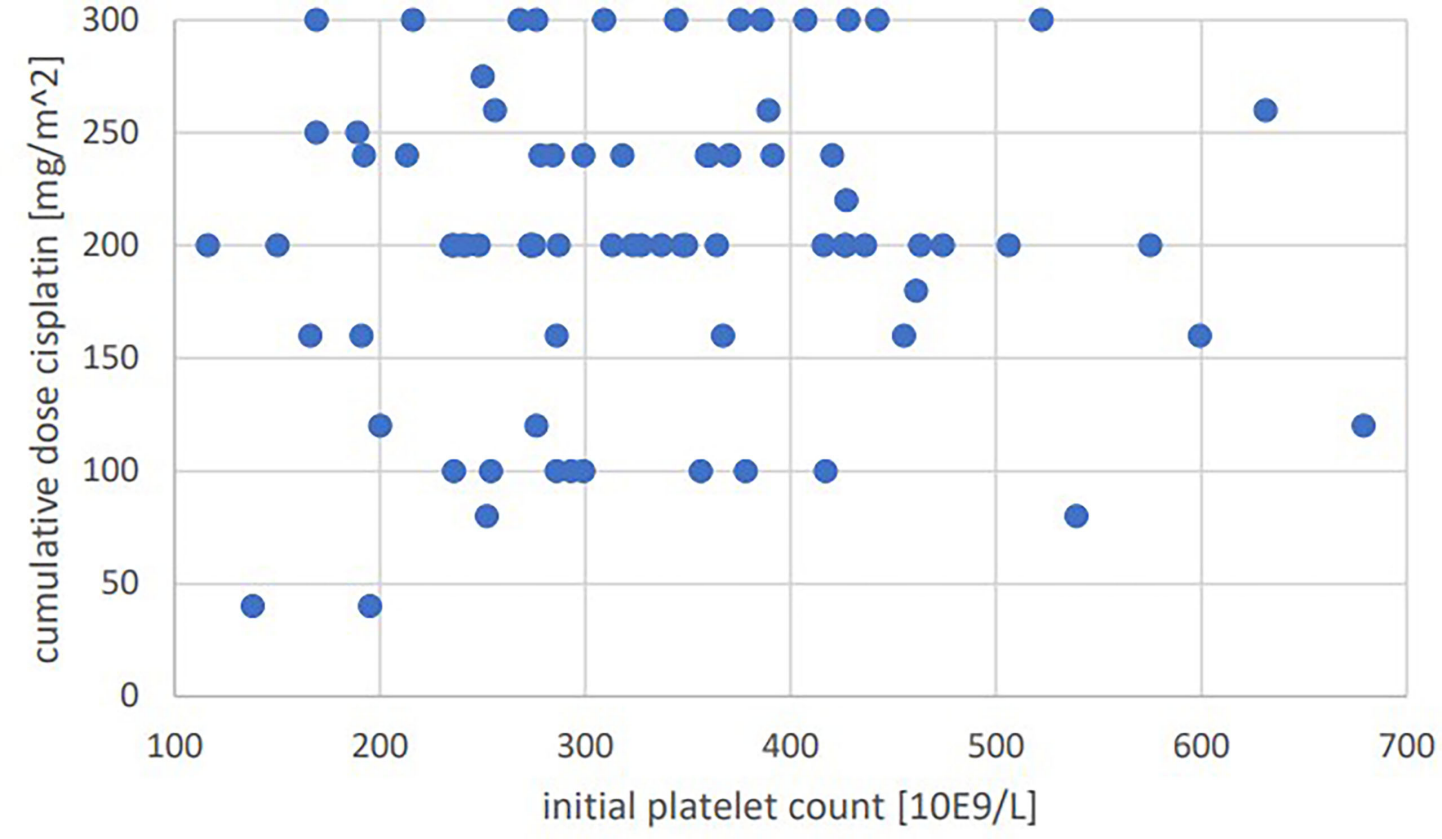
Bivariate correlation analysis of initial platelet count [10E9/L] with final cumulative total dose of cisplatin achieved [mg/m^2]: p=0.56, no significant correlation; individual values as points.

Significant positive correlations were seen for total cumulative dose with initial GFR (p=0.001, Pearson’s r=0.364) (**Figure 1**), initial hemoglobin (p=0.035, r=0.237) (**Figure 2**), initial erythrocyte count (p=0.002, r=0.337) (**Figure 3**), and initial albumin (p=0.002, r=0.337) (**Figure 4**). There were no significant correlations for total cumulative dose with initial leukocyte (**Figure 5**) or platelet (**Figure 6**) count. There were no significant correlations of the total cumulative dose with the differences of the laboratory values pre/post-chemo administration.

When separated by gender, there was a significant difference in the change in GFR after the first chemo administration, both in absolute (p=0.007) and relative differences (p=0.012), with a greater decrease in GFR in females (-7.27ml/min; -4.13ml/min). Otherwise, there were no significant differences, not even in the total cumulative dose achieved.

In the male subgroup, there was a significant negative correlation of total cumulative dose with an increase in leukocytes after the first chemo administration (absolute value: p=0.016, r=-0.301; relative value: p=0.032, r=-0.269).

In the subgroup of women, there was a significant negative correlation of differences in erythrocytes (absolute value: p=0.03, r=-0.708; relative value: p=0.04, r=-0.695) and hemoglobin (absolute value: p=0.028, r=-0.567; relative value: p=0.023, r=-0.581) values over the first chemo administration with the total cumulative dose.

Between patients with and without PEG, as well as between patients with and without port, there were no significant differences in the values collected.

**Figure 7:**
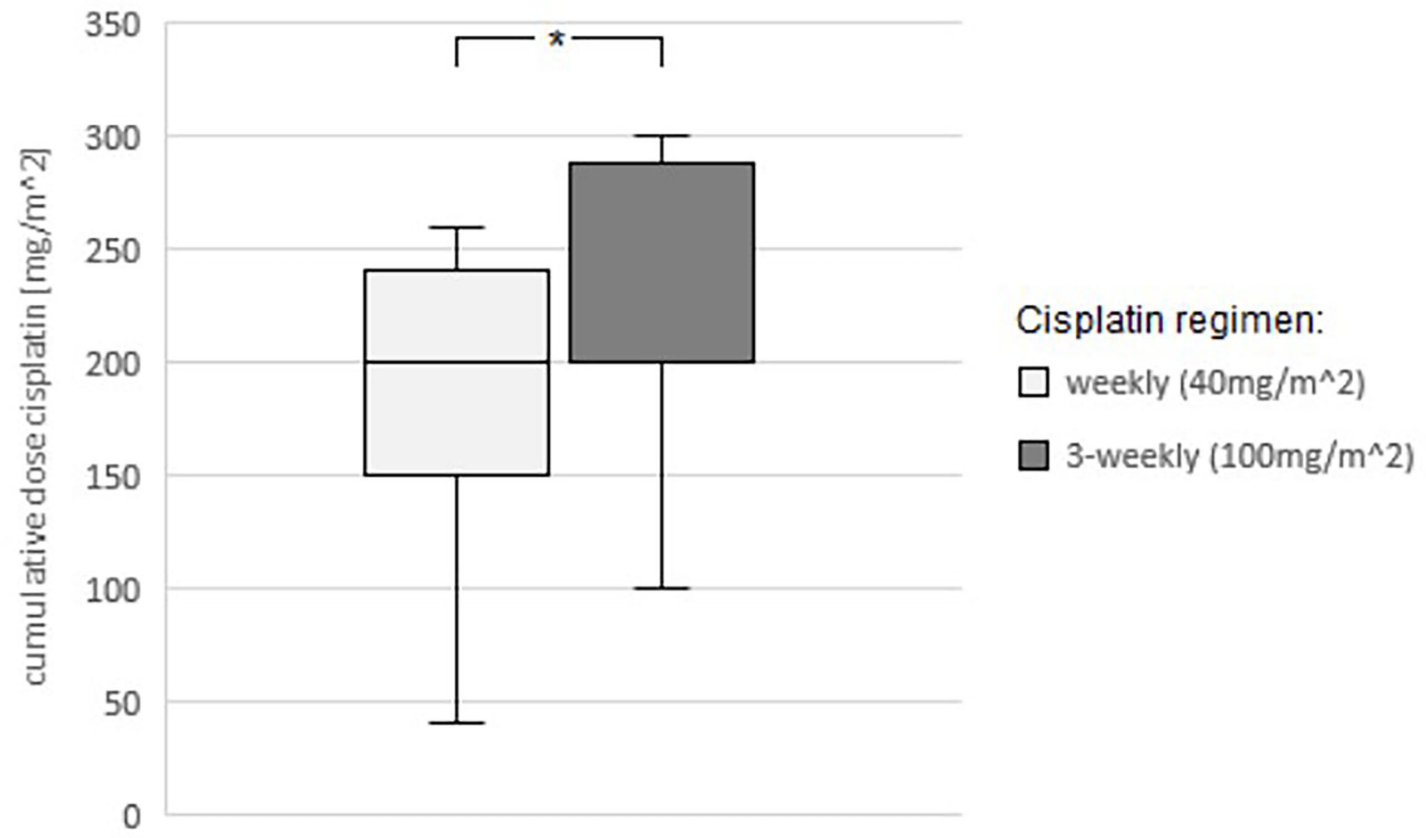
Comparison of the average cumulative total dose of cisplatin achieved between the two regimens (weekly administration 40mg/m^2 body surface area and 3-weekly administration 100mg/m^2 body surface area). Student’s t-test: p=0.046, statistically significant (*) mean CDC(weekly)= 183.33mg/m^2; mean CDC(3-weekly)=214.18mg/m^2.

In the weekly group, the mean age (69.53 ± 7.1 years) was significantly (p<0.001) higher than that in the 3-weekly group (58.94 ± 7.75 years). The achieved total cumulative dose of cisplatin was significantly (p=0.046) higher in the 3-weekly group than in the weekly group (214.18 ± 65.95 vs. 183.33 ± 65.2 mg/m^2^ body surface area) (**Figure 7**). Mean initial GFR was significantly (p<0.001) lower in the weekly group (79.9 ± 17.22ml/min) than in the 3-weekly group (94.45 ± 14.23). There were no significant differences in the remaining initial laboratory values between the two groups.

Considering only patients with a GFR ≥ 70ml/min, there is no significant difference in the total cumulative dose of cisplatin achieved between the weekly regimen (190 ± 57.82mg/m^2^ body surface area) and the 3-weekly regimen (217.28 ± 62.34mg/m^2^ body surface area). Patients with GFR between 60-69ml/min showed a significant difference (p=0.046) with 183.33 ± 65.2mg/m^2^ body surface area in the weekly regimen and 214.18 ± 65.95mg/m^2^ body surface area in the 3-weekly regimen. Patients with a GFR<60ml/min did not receive chemotherapy on the 3-weekly regimen during the screened period, so a comparison of the two regimens was not possible here.

Initial leukocyte values showed a significant positive correlation with leukocyte values after the first (p=0.009, r=0.292) and before the fifth (p=0.019, r=0.518) chemo administration. Initial GFR values showed a significant positive correlation with GFR values before the second (p<0.001, r=0.701), third (p<0.001, r=0.668), fourth (p<0.001, r=0.749), fifth (p=0.004, r=0.164) and sixth (p<0.001, r=0.886) chemo administration. Initial platelet values showed a significant positive correlation with platelet values before the second (p<0.001, r=0.537), third (p=0.001, r= 0.473), fourth (p<0.001, r=0.656), fifth (p=0.002, r=0.646) and sixth (p=0.01, r=0.709) chemo administration. Initial hemoglobin values showed a significant positive correlation with hemoglobin values before the second (p<0.001, r=0.751), third (p<0.001, r=0.512), fourth (p<0.001, r=0.786), fifth (p<0.001, r=0.758) and sixth (p=0.002, r=0.79) chemo administration. Initial erythrocyte values showed significant positive correlation with erythrocyte values before second (p<0.001, r=0.605), third (p<0.001, r=0.507), fourth (p<0.001, r=0.768), fifth (p<0.001, r=0.788) and sixth (p=0.002, r=0.795) chemo administration. Initial albumin values showed significant positive correlation with albumin values before second (p<0.001, r=0.667), third (p=0.012, r=0.372) and fourth (p=0.002, r=0.586) chemo administration.

With respect to the individual initial laboratory values among themselves, significant positive correlations were found between leukocytes and platelets (p=0.003; r=0.335), GFR and platelets (p=0. 025; r=0.252), hemoglobin level and erythrocytes (p<0.001; r=0.759), hemoglobin level and albumin (p<0.001; r=0.651), and erythrocytes and albumin (p<0.001; r=0.607).

There was a significant negative correlation between platelets and hemoglobin level (p<0.001; r=-0.415), as well as between platelets and albumin (p<0.001; r=-0.409) and platelets and erythrocytes (p=0.006; r=-0.304).

## Discussion

There are currently only a few randomized controlled trials comparing the two aforementioned chemo regimens. Tsan et al. described a higher average cumulative total dose of cisplatin achieved with the 3-weekly regimen and better tolerability than with the weekly regimen (17). Noronha et al. compared the 3-weekly regimen with weekly administration of 30mg cisplatin/m^2^ body surface area, again finding significantly better locoregional control in the 3-weekly group and recommending it should be preferred (15). In a meta-analysis, no superiority of the weekly regimen was found in terms of patient outcome, nor were there differences in prior therapy side effects, so the authors recommend the 3-weekly regimen (18). Another meta-analysis also found no difference in overall survival, but found the weekly regimen to be less myelotoxic and nephrotoxic, but associated with increased dysphagia and body weight loss (19). Retrospective evaluations also found a higher total cumulative dose of cisplatin with the 3-weekly regimen (16), but other authors described better tolerability in terms of less toxicity with the weekly regimen (20). In addition to the two regimens listed, other regimens (such as low-dose daily cisplatin (21, 22) also exist internationally, but these are not currently established nationwide in Germany. It is to be expected that the results of this study are not directly transferable to other cisplatin regimens and that own evaluations have to be made in this respect.

An important retrospective study, in light of whose results our study is also interpreted, showed a correlation of overall survival with the total cumulative dose of cisplatin administered (13). This study was important in that it was able to correlate a targeted but more distant end point (overall survival) with a more proximate end point (total cumulative dose) and therefore allowed earlier interpretation of data.

Due to the highly distressing and also potentially dangerous side effects of (radio)chemotherapy, it seems reasonable to consider not only tumor free-survival but also the overall survival of patients with regard to the evaluation of a therapy. However, due to the partly limited compliance of patients during regular tumor follow-up, the survival of all patients could not be evaluated. Of those who could be followed up, 26.6% of patients after RCT with the weekly regimen died within the observation period; in the 3-weekly regimen group, 8.1% of patients died within the observation period. The significance of the survival rates of our patients is therefore limited, also due to the relatively short observation period in relation to the 5-year survival rate of patients with HNSCC (23). Comparison of laboratory values at baseline showed significantly higher erythrocyte, hemoglobin, and albumin levels and lower platelet levels in survivors. Overall, however, it is difficult to compare patient survival between the two regimens in a retrospective study because the indication of the regimens was also based on the treating physician’s assessment of which form of chemotherapy the patient could tolerate. Based on the significant differences in erythrocyte/hemoglobin and albumin levels, it can be assumed that the patients who died during the observation period were already in poorer physical condition before the start of therapy, so that their poorer survival rate cannot necessarily be attributed to the chemo regimen. Thus, a high-quality assessment of overall survival comparing cisplatin regimens remains reserved for large, prospective studies designed over an even longer time period.

Based on the study by Strojan et al. the cumulative total dose was therefore set as the primary endpoint in this study.

There were no significant differences in the total cumulative dose achieved between patients with and without PEG, and patients with and without port. Dysphagia in patients with advanced HNSCC can develop both promptly postoperatively due to extensive tumor resection and persist in aRCT (24), or develop directly in a pRCT or aRCT due to radiogenic stomatitis and xerostomia (25). If oral food intake is significantly restricted as a result, the placement of a PEG may be necessary in the medium term after temporary parenteral nutrition. The patient must be informed about the possible risks and complications (26). If extravasation occurs during intravenous administration of cisplatin, severe irritation of the affected tissue, including necrosis, may occur, necessitating immediate conservative or even surgical intervention (27). Because of the higher concentration, the risk of tissue damage is higher in the 3-weekly dosage than in the weekly dosage (28). Therefore, pretherapeutic port implantation should be evaluated, especially in patients with poor peripheral venous status. However, due to the potential peri- and postoperative complications from a port, the expected benefits must always be weighed against the potential risks (29). Thus, the indication for the mentioned devices has to be made individually for the patients, a basic superiority in the achieved cumulative total dose could not be proven in our data by PEG placement and/or port implantation, a blanket recommendation on this can therefore not be made.

Our data show that high erythrocyte, hemoglobin, GFR, and albumin values in the initial blood values, i.e., measured before the first chemotherapy dose, correlate with a high total cumulative dose achieved.

High or normal hemoglobin values, like high or normal erythrocytes, indicate adequate hematopoietic function. A high or normal GFR indicates adequate renal function. Albumin, as the main blood transport protein, is an indirect representation of nutritional status and, in combination with other values, is known to be a predictive factor about patient outcome in long-term therapies (30).

It seems logical that high/normal values of leucocytes and platelets also indicate normal bone marrow function. The lack of correlations of leukocytes and platelets with the cumulative total dose of cisplatin in our data allows hypothesizing different explanations. On the one hand, these values in the context of reactive leukocytosis and reactive thrombocytosis are subject to higher dynamics with higher fluctuations within a few days than, for example, erythrocyte values. This makes them unsuitable for predictive estimation for the future based on a single blood draw. On the other hand, it could be suggested that the bone marrow function, which is represented by the leukocyte and platelet values, actually has no predictive/prognostic function for the patient. This is in contrast to the demonstrated correlations of hemoglobin and erythrocytes. In this regard, it could be argued that erythrocyte and hemoglobin levels do not primarily represent bone marrow function, but rather renal function. An adequate renal function plays an important role in erythrocyte formation by stimulating hematopoiesis *via* erythropoietin (31). Although this relationship is biologically beyond question, we were unable to demonstrate a significant correlation between GFR and erythrocyte/hemoglobin levels in our data. This suggests that erythrocytes are still influenced by too many other factors to assume a pure linear relationship with, and thus representation of, renal function. A final conclusion of this cannot be made in a retrospective view due to the close biological interconnectedness of the laboratory values.

In summary, patients with adequately functioning organ systems, particularly the kidney and bone marrow, have a better chance of achieving a high total cumulative dose of cisplatin than patients who already have laboratory limitations in these systems at the start of therapy. However, it must be kept in mind that although the positive correlations were statistically significant, they were with a rather moderate correlation coefficient, so the associations between good renal/bone marrow function and achieving a high total cumulative dose of cisplatin were not strictly related. Rather, the organ functions reflected in the blood values must be viewed and evaluated in conjunction with, for example, the patient’s clinical presentation and other comorbidities as well, not as sole decision factors.

High/normal initial values of hemoglobin, GFR, erythrocyte count and albumin can therefore be considered predictive factors for chemotherapy with cisplatin. Yet, there are also clear positive correlations within the laboratory values, especially strong between the hemoglobin values, the erythrocytes and the albumin values. Due to their biological nature, these values are closely related and also typically move in conformity with each other, as they represent the general condition as well as, among other things, the nutritional status of the patient. Malnutrition, for example, can manifest itself both in a decreased albumin level and, in the case of pronounced substrate deficiency, in anemia. It must therefore be assumed that the individual laboratory values are not completely independent values, each of which is individually predictive of the subsequent cumulative total dose, but must be considered in their entirety.

The importance of GFR as a prognostic factor is also highlighted by the fact that in the subgroup analysis of patients with a GFR of more than 70ml/min/1.73m^2, no significant difference in the cumulative dose of cisplatin was discernible between the 3-weekly and the weekly group. This highlights the importance of good renal function for the patient’s therapeutic prognosis and might even call into question the fundamental superiority of the 3-weekly regimen. Nonetheless, the superiority of this regimen in terms of higher cumulative dose of cisplatin has also been demonstrated in large randomized trials (15, 17), also in our study significantly higher cumulative doses were achieved with the 3-weekly regimen in the range between 60 and 69ml/min/1.73m^2.

However, it should be noted that in routine clinical practice the measurement is made indirectly by calculation from serum creatinine. Creatinine, in turn, is a breakdown product of muscle. Especially in (tumor) cachectic patients, creatinine may be decreased due to the lack of muscle mass, so that a false-high/good GFR value is calculated. In these cases, the renal function must be critically questioned.

In our data, there was a significant difference in the dynamics of GFR after the first chemo administration between men and women, with women showing a greater decrease in GFR. It is known from the analysis of long-term data that women show a significantly greater permanent decrease in GFR during the course after therapy with cisplatin compared to men (32). This is mainly explained by the lower average muscle percentage in women (33). Over the later chemo administrations, this sex difference in GFR dynamics was no longer detectable in our data, probably because the patients who had shown a greater GFR decline during the first administration had received targeted protective volume therapy during later chemo administrations to prevent renal failure.

The male subgroup showed a negative correlation of the dynamics of leukocyte levels over the first chemo administration with the later cumulative dose of cisplatin. Thus, the higher the leukocytes increased between before and after the first administration, the lower the cumulative dose of cisplatin at the end. It can be assumed that this correlation is due to constellations in which patients have suffered an infection, which on the one hand manifests itself in leukocytosis and on the other hand negatively influences the cumulative dose achieved later, since the infection meant that chemotherapy had to be suspended or discontinued. A possible correlation of leukopenia after cisplatin administration with the later cumulative dose may thus be statistically masked.

A similar negative correlation was found in the subgroup of women, but here between the dynamics of erythroycyte and hemoglobin levels above the first chemo administration with the cumulative dose of cisplatin. Thus, the greater the increase in erythrocyte and hemoglobin levels between the first two blood draws, the lower the subsequent cumulative dose of cisplatin. Since high erythrocyte and hemoglobin levels tend to be prognostically favorable factors, this counterintuitive correlation is most likely explained by an exciccosis phenomenon. Renal damage and limited fluid intake may result in intravascular fluid deficiency, which is reflected in increased erythrocyte and hemoglobin levels. Since this correlation in our data is only seen in the first chemo administration, it can be assumed that in these patients exsiccosis was counteracted by supportive measures during later chemo administrations.

The correlation analyses show that GFR, platelet count, erythrocyte count, and hemoglobin levels are stable overall, and the initial values correlate with the later ones over almost the entire course of chemotherapy. In the case of leukocyte values, a consistent correlation of initial with later values is not found, which fits with the fact that leukocytosis may occur together with an increase in acute-phase proteins (34) in the context of stressful situations such as those represented by chemotherapy. At the same time, however, leukopenia may also develop due to the myelosuppressive effects of cisplatin (12). Through this, the dynamics of leukocyte values in the course of chemotherapy is much more pronounced than that of the other laboratory values, resulting in the lack of correlation evidence with the initial values. However, there was no relevant correlation of the initial laboratory values with the later dynamics of the laboratory values, nor of the dynamics of the laboratory values over the first chemo administration with the later values. It must also be considered that falsification may occur due to iatrogenic influence on laboratory parameters during the course of radiochemotherapy. While cisplatin can primarily trigger a reduction in blood count values, the treating physician can raise blood count values again by administering transfusions and bone marrow stimulation with e.g. granulocyte colony-stimulating factor (G-CSF). Substitution therapy of iron and erythropoietin in the context of anemia of chronic disease also changes the course of blood values compared to a patient in whom this substitution does not take place.

It must further be considered that radiotherapy alone at different doses also has an impact on blood counts and blood values and thus represents a potential bias on the evaluation of blood values under radiochemotherapy (35). However, in our study, significant correlations and differences were found mainly with respect to initial laboratory values, which were not yet influenced by radiotherapy, and after the first dose of chemotherapy, which was administered in parallel with the start of radiotherapy. Thus, the influence of radiation on the significant correlations and differences found can be considered absent or negligible. The later laboratory values in the course of radiochemotherapy were certainly influenced by the radiation in addition to the chemotherapy; an etiological assignment would not be possible with certainty here in the retrospective design.

In summary, our data show that neither the initial laboratory values nor their dynamics over the first chemo administration provide sufficient information about the later behavior of the laboratory values in the course of chemotherapy. Thus, subsequent leukopenia or renal failure cannot be confidently assessed after primary chemo administration and need to be monitored regularly during chemotherapy. Nevertheless, it must be critically noted that the sampling times of the blood controls after chemo administration were not standardized. Due to the fluctuations in the sampling times, in some cases of several days, a falsification of the dynamics cannot be ruled out here. In clinical routine, the partly specific temporal course of the laboratory parameters, in particular of the nadir, should be observed during the blood checks after chemo administration in order not to obtain false-good values.

However, the negative correlation of the initial platelet values with the initial erythrocyte, hemoglobin, and albumin values, i.e., the laboratory values favorable in relation to the cumulative total dose, is striking. A direct negative correlation between the initial or later platelet values and the total cumulative dose of cisplatin was not detectable in our data. However, advanced cancers are well known causes of secondary thrombocytosis (36), and the negative prognostic value of thrombocytosis and an elevated platelet/lymphocyte ratio in HNSCC patients has already been demonstrated in other retrospective studies (37). Although the value in terms of patient outcome of antiplatelet therapy (38) is not yet clear, pretherapeutic thrombocytosis before cisplatin therapy seems to have some prognostic value.

The limitations of this study are mainly due to its retrospective design. As the initial regimens were also chosen on the basis of the initial laboratory values, an asymmetry in the weekly and 3-weekly groups results, especially with regard to age and GFR. A younger patient with statistically less preexisting disease and good renal function was likely to be preferentially assigned to the 3-weekly regimen by the treating physician, whereas an older patient with statistically more preexisting internal disease and a low GFR was more likely to be placed in the weekly group. However, our results are in line with those of the literature, in which a higher cumulative total dose was achieved in the study arm with the 3-weekly regimen, even in randomized controlled trials (15, 17). Furthermore, the changes in laboratory values over the period of the RCT cannot necessarily be attributed to the chemotherapy, as iatrogenic interventions such as red blood cell transfusions or the administration of G-CSF could also have taken place. However, since these are legitimate supportive measures that are available to every patient under RCT and would not have been omitted in a prospective study, the use of the data is nevertheless justifiable. In particular, initial blood levels, for which we demonstrated a correlation with the cumulative total dose of cisplatin, are still unaffected by such supportive measures.

Our data show a higher average cumulative total dose of cisplatin in the 3-weekly group (**Figure 7**). Also, a cumulative dose ≥200mg/m^2^ body surface area was more frequently achieved with the 3-weekly regimen. If the previous findings from retrospective studies confirm that the cumulative total dose correlates positively with the outcome of the patients, the 3-weekly would in principle be preferable to the weekly regimen as far as medically justifiable.

The initial hemoglobin and erythrocyte values, the initial GFR and the initial albumin value allow a prospect of the total dose to be achieved later and should be taken into account when selecting the chemo regimen. Also, elevated platelet values are seen mainly in patients with otherwise rather low favorable prognostic parameters (erythrocytes/hemoglobin/albumin), so that at least indirectly an increased disease burden and a lower resistance might be suspected here.

The initial laboratory values as well as their changes after the first chemo administration do not allow any conclusion to be drawn about later changes in the laboratory values (especially leukopenia or kidney failure), therefore the laboratory values must be checked regularly during the entire chemotherapy in order to be able to recognize and treat any complications at an early stage.

However, rising leukocytes (mainly in men in our evaluation) and rising erythrocytes/hemoglobin (mainly in women in our evaluation) after the first chemo administration may be indications of poor outcome, as they were statistically associated with a lower cumulative dose of cisplatin in our study.

For the correct selection of the chemo regimen, especially against the background of cisplatin-resistant tumors, further factors must be investigated in the future in order to be able to make the optimal weighing of benefits and risks for the individual patient. In this regard, tumor biology and pharmacogenetic research are the main focus for patient stratification (39). This will allow the identification of patient groups with an increased risk of complications and side effects, as well as those with a possible development of resistance to cisplatin.

## Data Availability Statement

The raw data supporting the conclusions of this article will be made available by the authors, without undue reservation.

## Ethics Statement

The studies involving human participants were reviewed and approved by Ethik-Kommission II of the University Heidelberg. Written informed consent for participation was not required for this study in accordance with the national legislation and the institutional requirements.

## Author Contributions

FJ formulated the hypothesis, conducted the data analysis and literature review, and wrote the manuscript. AL supervised the writing of the manuscript and revised it. LH, SL, NR, BW, and LZ revised and corrected the manuscript and made essential suggestions for adaptation and modification. Each author made an essential contribution to the form and content of the current manuscript.

## Conflict of Interest

The authors declare that the research was conducted in the absence of any commercial or financial relationships that could be construed as a potential conflict of interest.

## Publisher’s Note

All claims expressed in this article are solely those of the authors and do not necessarily represent those of their affiliated organizations, or those of the publisher, the editors and the reviewers. Any product that may be evaluated in this article, or claim that may be made by its manufacturer, is not guaranteed or endorsed by the publisher.
